# Racial variations in maxillomandibular advancement for obstructive sleep apnea: a systematic review and meta-analysis

**DOI:** 10.1007/s11325-024-03211-0

**Published:** 2024-12-09

**Authors:** Douglas P Nanu, Tanner J. Diemer, Shaun A. Nguyen, Timothy Tremont, Ted A. Meyer, Mohamed Abdelwahab

**Affiliations:** 1 Department of Otolaryngology-Head and Neck Surgery, Medical University of South Carolina, Charleston, SC USA; 2 Elson S. Floyd College of Medicine at Washington State University, Spokane, WA USA; 3 University of Arizona College of Medicine-Phoenix, Phoenix, AZ USA; 4 Department of Orthodontics, Medical University of South Carolina, Charleston, SC USA

**Keywords:** Maxillomandibular surgery, Orthognathic surgery, OSA, Sleep apnea, Race, Ethnicity

## Abstract

**Purpose:**

We aimed to explore alterations in polysomnographic, cephalometric, and subjective outcomes amongst different ethnic/racial groups after MMA for OSA.

**Methods:**

A meta-analysis was performed according to PRISMA reporting guidelines. The COCHRANE Library, CINAHL, PubMed, and Scopus were searched from inception to August 8, 2023. Each measure was weighted according to the number of patients affected. Heterogeneity among studies was assessed using χ2 and I2 statistics with fixed effects (I^2^ < 50%) and random effects (I^2^ ≥ 50%).

**Results:**

Twenty studies with a total of 469 patients (*n* = 257 Caucasians, *n* = 204 Asians, *n* = 8 Latinos) with a mean patient age of 40.0 years (range: 18–67; 95% CI: 38.0, 42.1). The mean difference for Caucasians were AHI: -39.6 (95% CI: -55.0, -24.1; *p* <.001), LSAT: 7.5 (95% CI: 5.7, 9.3; *p* <.0001), and ESS: -4.5 (95% CI: -5.6, -3.4; *p* <.0001). The mean difference for Asians were AHI: -42.7 (95% CI -49.3, -36.0; *p* <.0001), LSAT: 13.8 (95% CI: 10.0, 17.4; *p* <.0001), and ESS: -6.7 (95% CI: -9.9, -3.5; *p* <.0001). The mean difference for Latinos were AHI: -21.2 (95 CI%: -37.7, -4.6; *p* =.01), and ESS: -2.0 (-7.9, 3.9; *p* =.50). Greater reduction of AHI was seen in Asians vs. Caucasians and Asians vs. Latinos. Lastly, the reduction of ESS was significantly better for Asians vs. Caucasians.

**Conclusion:**

The study highlights significant variations in MMA outcomes among different ethnic/racial groups. Asians tend to have more severe OSA preoperatively and experience greater postoperative improvements in AHI, LSAT, and ESS compared to Caucasians.

**Supplementary Information:**

The online version contains supplementary material available at 10.1007/s11325-024-03211-0.

## Introduction

Obstructive sleep apnea (OSA) is a common sleep-related breathing disorder that affects 5 to 24% of the adult population and has a higher incidence among males [1–3]. It is characterized by interruptions in the airflow through the upper airway resulting in nocturnal hypoxemia. Long-term outcomes of this hypoxemia vary from increased risk of cardiovascular morbidity and mortality to endocrinal and neurological deterioration with subsequent increases in healthcare utilization [4–6]. Thus, managing OSA is essential for minimizing long-term health risks. While continuous positive pressure (CPAP) is the gold standard treatment, not all patients adhere to this treatment, and some cannot tolerate it. In such instances, surgical interventions are indicated, including maxillomandibular advancement (MMA) [7]. Maxillomandibular advancement is a highly effective surgical option that relieves airway collapse by expanding the skeletal framework (Appendix 1) [8, 9]. While MMA can be associated with risks of malocclusion, paresthesia, tingling, and possible relapse, major complications are extremely rare [8]. It is especially indicated for patients with moderate to severe OSA who do not respond adequately to CPAP therapy or other non-surgical or surgical treatments. A Le Fort I osteotomy is typically performed along with bilateral sagittal split osteotomies (BSSO) to reposition/advance the maxillomandibular complex [7].

Prior meta-analyses have conclusively shown that MMA surgery significantly reduces the apnea-hypopnea index (AHI), with a surgical success rate of 85.0–100%, even amongst the morbidly obese (surgical success rate of 85.3%) [8, 10–12]. Maxillomandibular advancement surgery also showed efficacy in patients with craniofacial/maxillomandibular abnormalities (such as dentofacial class 2 and 3). Class 2 patients, despite having more severe preoperative OSA compared to class 3, showed marked improvement. MMA was effective in both dentofacial class 2 and 3 groups. Both objective measures (e.g., Apnea-Hypopnea Index [AHI], Oxygen Desaturation Index [ODI]) and subjective outcomes (e.g., patient-reported quality of life, sleep quality) show significant improvements in these groups [9].

While the literature supports the effectiveness of MMA surgery, there is a scarcity of data regarding racial and ethnic outcomes post-MMA [13–15]. Studying racial surgical and cephalometric outcomes is vital to addressing healthcare disparities and personalizing treatments to individual patient groups [16, 17]. The hypothesis is that there is a discrepancy between OSA patients of different racial/ethnic groups in their polysomnographic and cephalometric measures. The aim of this study is to evaluate variabilities and outcomes across different racial and ethnic groups undergoing MMA for OSA and identify potential factors impacting these findings.

## Methods

### Study selection

This study was conducted according to Preferred Reporting Items for Systematic Reviews and Meta-Analysis (PRISMA) guidelines [18]. Two researchers (D.N. and T.D.) independently performed a literature search to identify potentially relevant studies via Scopus, PubMed, CINAHL, and Cochrane libraries. For a study to be included in this review for analysis, (1) subjects had to have undergone MMA for OSA, (2) pre- and postoperative objective quantitative outcomes needed to be reported for at least one of the following: AHI, ESS, LSAT, Sella nasion point A (SNA) angle, Sella nasion point B (SNB) angle and (3) racial data needed to be reported in the article or via email request to the corresponding author; (4) studies needed to be in English. The SNA (Sella, nasion and A point) angle measures the anteroposterior position of the maxilla relative to the anterior cranial base. The SNB (Sella, nasion and B point) angle measures the anteroposterior position of the mandible relative to the anterior cranial base. We excluded (1) studies without racial data reported in the article or via email request to the corresponding author, (2) studies that included patients under the age of 18 years old, (3) studies that included syndromic patients; (4) studies that were case reports, discussions, editorials, book chapters, commentary, and systematic reviews with or without meta-analysis. Search strategies can be found on Appendix 2.

### Data extraction

Data collected from each study were extracted into a spreadsheet (Excel 2023; Microsoft Corporation). Extracted data included nominal data such as the total number of patients, gender (males and females), the total number of individuals based on race (Caucasians, Asian, and Latino), and the total number of surgical successes. Continuous data extracted included age, BMI, AHI, ESS, LSAT, SNA, SNB, mandibular and maxillary advancement lengths.

### Methodological quality of included studies

Racial groups were selected based on established racial categories according to the United States Census Bureau (i.e. Caucasian, Asian, Latino, Black) [19]. Studies included consisted of 20 case-series. We screened 1578 MMA studies for potential relevance via Covidence (Covidence systematic review software, Veritas Health Innovation, Melbourne, Australia); 168 nonduplicated full-text studies were assessed for eligibility (Appendix 3). English studies were included. After reviewing 168 full-text studies, 96 studies were to be included; however, not all the studies contained clear racial demographic data. An email was sent out to the corresponding authors requesting racial demographic data. One author out of 53 authors replied via email with viable racial demographic data [20]. Two included studies had demographic data available upon request from the senior author (M.A.W) [9, 21]. A total of 20 case-series were included in the analysis. The Joanna Briggs Institute (JBI) Critical Appraisal Checklist for Case Series was used to assess case series risk of bias.^17^ The articles were reviewed independently by the first 2 authors using the checklists and rated each item as “yes,” “no,” “unclear,” or “not applicable.” All disagreements were resolved by a third author, S.A.N. Each item was given a score of “1” for “yes” and “0” for “no,” “not applicable,” or “unclear.” The case series checklists were scored out of 10. A score of 5 or higher on either checklist was considered at low risk for bias and was, therefore, included in the paper (Appendix 4) [22].

### Statistical analysis

Meta-analysis of continuous measures (age, BMI, follow-up time, and mandibular/maxillary advancements) and meta-analysis of mean difference (pre-treatment vs. post-treatment) was performed with Cochrane Review Manager (RevMan) version 5.4 (The Cochrane Collaboration 2020, United Kingdom). Meta-analysis of proportions were performed using MedCalc 20.305 (MedCalc Software, Ostend, Belgium). Each measure (mean/mean difference (Δ)/proportion (%)/ANOVA and 95% confidence interval (CI) was weighted according to the number of patients affected. As some studies reported the outcomes in median (first quartile, third quartile), the quantile estimation (QE) method was deployed to calculate the pooled estimates [23]. Heterogeneity among studies was assessed using χ2 and I^2^ statistics with fixed effects (I^2^ < 50%) and random effects (I^2^ ≥ 50%). In addition, a comparison of proportions was done to compare racial/ethnic groups. In addition, significant differences with objective parameters (AHI, LSAT, SNA, SNB) and subjective parameters (ESS) among the 4 groups (Overall, Caucasian, Asian, Latino) were analyzed using a weighted one-way analysis of variance (ANOVA). In cephalometry, SNA and SNB is used as a metric for evaluating the sagittal position of the maxilla and mandible respectively. Further analyses were performed using post-hoc Tukey’s for comparison between groups. Finally, potential publication bias was evaluated by visual inspection of the funnel plot and Egger’s regression test, which statistically examines the asymmetry of the funnel plot (Appendix 5) [24, 25]. A *p*-value of < 0.05 was considered significant for all statistical tests.

### Outcome measures

The primary outcomes were polysomnographic data AHI, SpO2 levels, surgical success, LSAT and AHI percent reduction. Secondary outcomes were ESS, SNA, SNB, and advancements in mm (maxillary and mandibular) amongst different racial/ethnic groups. Surgical success was defined as an AHI of less than 20 events per hour and a 50% reduction in AHI levels post-MMA surgery [25]. Studies without pre- and post-operative values for at least one of these outcomes were excluded.

## Results

Individual data from 20 studies including 469 unique patients all undergoing MMA surgery for OSA were extracted [9, 20, 21, 27–43]. Studies included for analysis were published from 2008 to 2023 and originated from eight different countries. All studies included in this review were level 4 Oxford Level of Evidence. The JBI critical appraisal indicated an overall acceptable low risk of bias for all case-series (Appendix 4). A funnel plot (Appendix 5) with Egger’s test (74.1%, 95% CI: 59.8 to 83.3; *p* =.59) suggested little publication bias. Of the 13 studies that reported BMI values, the ΔBMI was found to be 0.4 (95% CI: -0.2 to 0.9; *p* =.17). Out of 469 patients, 52.4% (95% CI: 24.5 to 79.5) were Caucasian, 46.0% (95% CI: 19.0 to 74.4) were Asian, and 1.8% (95% CI: 0.8 to 3.5) were of Latino origin. The mean patient age was 40.0 years (range: 18–67, 95% CI: 38.0 to 42.1). Male and female distribution across all 20 studies was 83.1% (95% CI: 76.0 to 89.3) and 16.9% (95% CI: 10.7 to 24.0) respectively. The mean follow-up time for post operative studies was 8.0 months (range: 1.0–24.0 months) [9, 21, 27–38, 40–43]. The overall surgical success was 88.1% which was either reported in the study or was calculated using individual patient data [9, 20, 29, 30, 33, 35–41, 43]. For the Caucasian, Asian, and Latino groups, surgical success was 83.2%, 93.5%, and 100.0% respectively. The mean follow-up time for post operative studies was 8.0 months (range: 1.0–24.0 months) [9, 21, 27–38, 40–43]. The overall surgical success was 88.1% which was either reported in the study or was calculated using individual patient data [9, 20, 29, 30, 33, 35–41, 43]. For the Caucasian, Asian, and Latino groups, surgical success was 83.2%, 93.5%, and 100.0% respectively. Among 11 studies, the overall mean maxillary advancement was 5.6 mm (95% CI: 3.9 to 7.5). For Caucasians, it was 7.1 mm (95% CI: 5.2 to 9.0) [20, 29, 31, 33, 38, 39], and for Asians, was 3.7 mm (95% CI: 1.5 to 5.9) [32, 35, 41–43]. Maxillary advancement in Caucasians was significantly greater than Asians (*p =* .02). Among the same 11 studies, the overall mean mandibular advancement was 10.1 mm (95% CI: 8.8 to 11.4). For Caucasians, it was 9.9 mm (95% CI: 7.9 to 11.8) [20, 29, 31, 33, 38, 39], and for Asians, was 10.5 mm (95% CI: 8.6 to 12.3) [32, 35, 41–43], with no significant difference in mandibular advancement between them (*p =* .66). There was no maxillary or mandibular advancement data for the Latino group. A summary of patient characteristics and included studies can be found in Table 1.

**Table 1:** Study characteristics

Study	Country	*N*	Race	Mean Follow Up (Range)	Previous Interventions
S1	USA	28	Caucasian, Asian and Latino	6 mo	CPAP, Nasal septoplasty
S2	USA	28	Caucasian, Asian and Latino	6 mo	CPAP
S3	France	20	Caucasian	6 mo	UPPP, GAHT, CPAP
S4	Italy	48	Caucasian	6 mo	Unknown
S5	Canada	21	Caucasian	6 mo	Unknown
S6	Singapore	11	Asian	7.7 mo	Unknown
S7	Spain	25	Caucasian	9 mo	Unknown
S8	Korea	14	Asian	6 mo	CPAP
S9	Italy	25	Caucasian	1 mo	CPAP
S10	Taiwan	20	Asian	14 (6–27) mo	CPAP, unspecified surgery
S11	Taiwan	53	Asian	12 mo	CPAP
S12	Taiwan	12	Asian	3 mo	CPAP, soft tissue surgery
S13	China	12	Asian	6 mo	UPPP
S14	USA	15	Caucasian	6 mo	CPAP
S15	Belgium	32	Caucasian	Unknown	CPAP
S16	USA	24	Caucasian	6.7 mo	UPPP, GA, nasal septoplasty, bilateral tonsillectomy
S17	Italy	10	Caucasian	12 mo	Unknown
S18	China	33	Asian	12 mo	UPPP, CPAP
S19	China	28	Asian	12 > mo	CPAP
S20	China	10	Asian	24 mo	CPAP

S1: Abdelwahab A 2023, S2: Abdelwahab B 2023, S3: Bettega 2000, S4: Brevi 2015, S5: Curran 2022, S6: Goh 2003, S7: Gonzalez 2020, S8: Jeong 2017, S9: Lagana 2023, S10: Liao 2015, S11: Lin 2020, S12: Lin 2011, S13: Liu 2012, S14: Lye 2008, S15: VanderCruyssen 2019, S16: Verghese 2012, S17: Verze 2017, S18: Wei 2017, S19: Wu 2019, S20: Yu 2017

### AHI (427 patients in 18 studies) [9, 20, 27–31, 33–43]

**Fig. 1:**
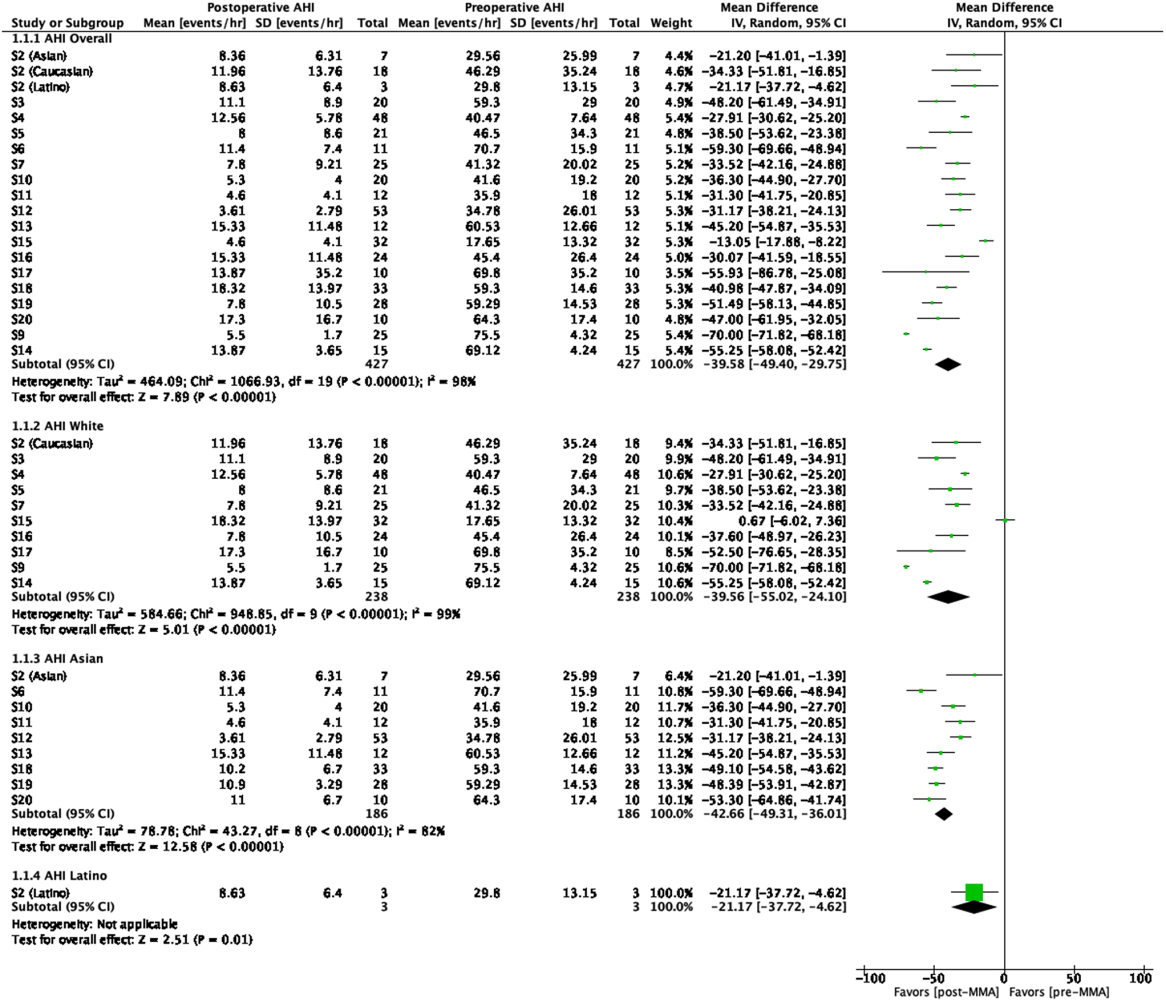
AHI mean differences

For AHI outcomes, ten studies evaluated Caucasian patients [9, 20, 27–29, 31, 33, 36–40], nine evaluated Asian patients [9, 30, 34–37, 41–43], and one evaluated Latino patients [9]. A meta-analysis was conducted to assess the mean difference (Δ) in AHI between pre-MMA and post-MMA surgeries. The overall group exhibited a significant improvement, with a ΔAHI of -39.6 (95% CI: -49.4 to -29.8; *p* <.001). The ΔAHI was − 39.6 (95% CI: -55.0 to -24.1; *p* <.00001) for the Caucasian group, -42.7 (*p* <.00001) for the Asian group, and − 21.2 (95% CI: -37.7 to -4.6; *p* =.01) for the Latino group (Fig. 1). All racial groups demonstrated significant improvements in AHI scores, both individually and as a collective group. However, Asians showed greater AHI percent reduction (*p <.*001) compared to Caucasians and Latinos (Table 2).

**Table 2:** ANOVA of subjective, objective (polysomnographic) and cephalometric MMA surgery outcomes

		Overall Mean (SD)	Caucasian Mean (SD)	Asian Mean (SD)	Latino Mean (SD)	Comparison Groups	*P* value (Tukey’s HSD) *
AHI	Preop	47.8 (18.43)	47.26 (18.43)	48.8 (19.09)	29.8 (25.99)	Caucasians, Asians and Latinos	Caucasian vs. Asian = **0.8323** Caucasian vs. Latino = **0.3700** Asian vs. Latino **= 0.3399**
	% Reduction	-74.94	-67.56	-84.45	-71.04	Caucasians and Asians	*<0.0001
	Postop	9.82 (7.07)	11.32 (8.73)	7.92 (4.95)	8.63 (8.9)	Caucasians and Asians	*<0.0001
	***P*** **value**	**< 0.00001**	**< 0.00001**	**<0.00001**	**0.01**	**—**	**—**
	**N**	**427**	**238**	**186**	**3**	**—**	**—**
LSAT	Preop	76.25 (10.14)	80.47 (8.22)	73.8 (11.25)	**—**	Caucasians and Asians	*<0.0001
	Delta	11.07 (2.4)	7.47 (2.23)	13.77 (2.49)	**—**	Caucasians and Asians	*<0.0001
	Postop	87.76 (5.00)	87.33 (5.28)	88.02 (4.84)	**—**	Caucasians and Asians	**0.6006**
	***P*** **value**	**< 0.00001**	**< 0.00001**	**<0.00001**	**—**	**—**	**—**
	**N**	**210**	**77**	**133**	**—**	**—**	**—**
SNA	Preop	80.77 (3.43)	80.44 (3.49)	81.12 (3.38)	**—**	Caucasians and Asians	**0.3007**
	Delta	4.77 (1.60)	5.53 (1.63)	3.88 (1.57)	**—**	Caucasians and Asians	*<0.0001
	Postop	85.78 (3.67)	86.14 (3.67)	85.39 (3.67)	**—**	Caucasians and Asians	**0.2784**
	***P*** **value**	**<0.00001**	**< 0.00001**	**< 0.00001**	**—**	**—**	**—**
	**N**	**224**	**115**	**109**	**—**	**—**	**—**
SNB	Preop	74.78 (3.78)	76.09 (3.82)	73.39 (3.74)	**—**	Caucasians and Asians	*<0.0001
	Delta	5.12 (1.60)	4.8 (1.58)	5.46 (1.62)	**—**	Caucasians and Asians	*=0.006
	Postop	79.9 (3.42)	80.89 (3.36)	78.85 (3.48)	**—**	Caucasians and Asians	*<0.0001
	***P*** **value**	**<0.00001**	**< 0.00001**	**<0.00001**	**—**	**—**	**—**
	**N**	**224**	**115**	**109**	**—**	**—**	**—**
ESS	Preop	11.5 (4.32)	10.29 (5.14)	12.38 (3.8)	9.25 (4.13)	Caucasians, Asians and Latinos	Caucasian vs. Asian **= 0.0006** Caucasian vs. Latino **= 0.9151** Asian vs. Latino = **0.1949**
	Delta	-5.93 (1.66)	-4.53 (1.78)	-6.65 (1.58)	-5.92 (1.55)	Caucasians, Asians and Latinos	Caucasian vs. Asian **< 0.0001** Caucasian vs. Latino **= 0.1003** Asian vs. Latino = **0.6142**
	Postop	6.48 (3.19)	5.84 (3.00)	7.24 (3.37)	2.62 (2.64)	Caucasians, Asians and Latinos	Caucasian vs. Asian **= 0.0024** Caucasian vs. Latino **= 0.0317** Asian vs. Latino **= 0.0004**
	***P*** **value**	**<0.00001**	**< 0.00001**	**<0.0001**	**0.11**	**—**	**—**
	**N**	**284**	**108**	**168**	**8**	**—**	**—**

*Tukey’s honest significant difference test post-hoc significant findings between groups

When comparing AHI among ethnic/racial groups, there was an overall significant difference (*p* <.0001) in AHI values between at least two racial groups (Table 2). Further multiple comparisons between groups showed that the mean change (Δ) in AHI was greater in Asians vs. Caucasians at Δ -16.9 (95% CI: -20.4 to -13.3; *p <*.0001). Lastly, Asians had a lower (better) post-operative AHI value by -3.4 (95% CI: -5.2 to -1.6; *p* <.0001) compared to Caucasians.

### ESS (284 patients in 11 studies) [9, 20, 21, 29, 34–35, 39, 41, 42]

**Fig. 2:**
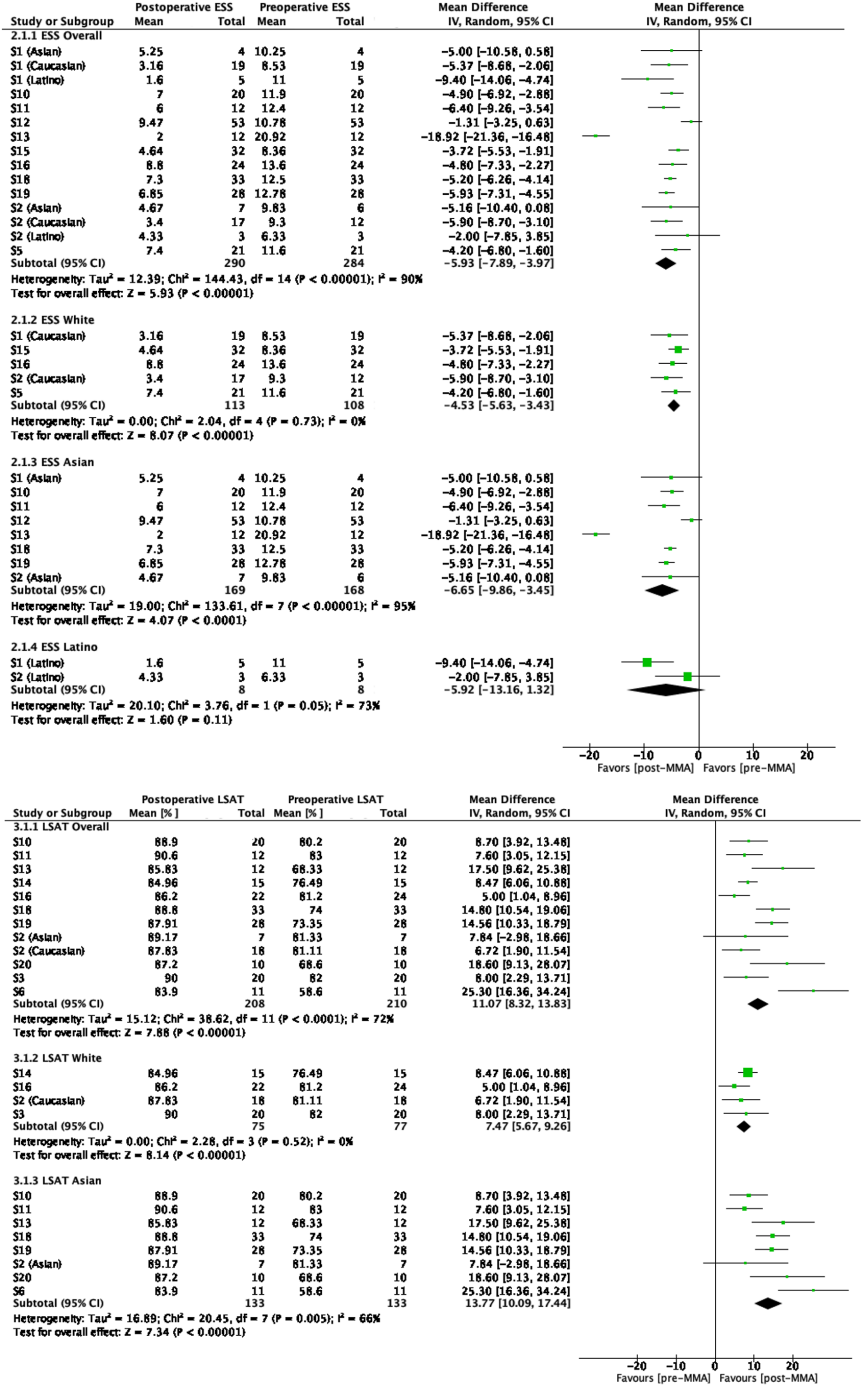
ESS and LSAT mean differences

Overall, there was a significant improvement in ESS (*p* <.00001), indicating an improvement in sleepiness post-MMA [44]. Improvements in the ESS were seen in the Caucasian (*p* <.00001) and Asian groups (*p* <.0001), while the Latino group did not reach significanc (Fig. 2).

Mean reduction in ESS was greater (better) amongst Asians compared to Caucasians at a Δ-2.1 (95% CI: -2.6, -1.6; *p <*.0001). Additionally, Asians had a greater reported ESS before and after MMA surgery compared to Caucasians (*p* <.0001, Table 2).

### Lowest O2 saturation levels (208 patients in 11 studies) [9, 27, 30, 34, 36–39, 41–43]

Overall, there was a significant improvement, with a ΔLSAT of 11.1 (95% CI: 8.3 to 13.8; *p* <.00001) (Fig. 2). For Caucasians, the ΔLSAT was 7.5 (95% CI: 5.7 to 9.3; *p* <.00001), for Asians, it was 13.8 (95% CI: 10.1 to 17.4; *p* <.00001). There were no data on LSAT values Latinos.

For LSAT, there was also a significant difference between racial groups (Table 2). The mean difference of LSAT was significantly greater in Asians than in Caucasians by Δ6.3 (95% CI: 5.5 to 7.1; *p <*.0001). Additionally, pre-operative LSAT values were lower (worse) pre-operatively amongst Asians compared to Caucasians by -6.7 (95% CI: -10.1 to -3.2; *p <*.0001), yet without difference in post-operative LSAT values (*p* =.60).

### Cephalometric analysis (224 patients in 10 studies) [20, 28, 32, 33, 34, 37, 40, 41–43]

**Fig. 3:**
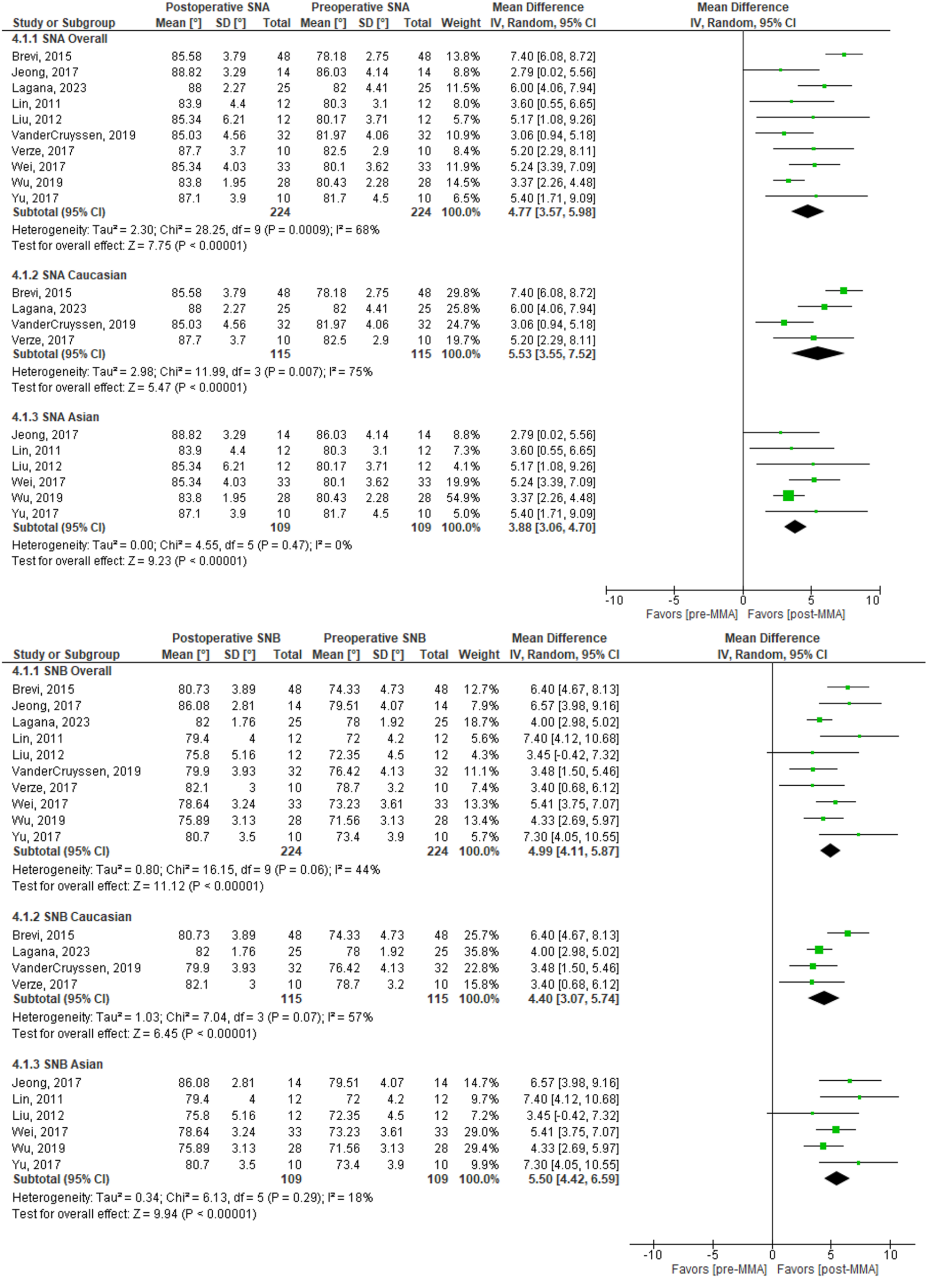
SNA and SNB mean differences

For SNA and SNB angles, 4 studies evaluated Caucasian patients [20, 28, 33, 40], and 6 evaluated Asian patients [32, 36, 37, 41–43]. Overall, there was an increase in SNA and SNB, with a ΔSNA of 4.8 (95% CI: 3.6 to 6.0; *p* <.00001) and ΔSNB of 5.0 (95% CI: 4.1 to 5.9; *p* <.00001). Results for the overall and individual racial groups can be found in Fig. 3.

There was a significant mean difference in SNA values between different racial/ethnic groups (Table 2). The mean difference of SNA was significantly higher in Caucasians than Asians by -1.7 (95% CI: -2.2 to -1.1; *p <*.0001). There was no significant difference in the pre-operative or postoperative SNA values between Caucasians and Asians (*p =*.30 and *p* =.28, respectively).

Additionally, the mean difference of SNB was higher in Asians than in Caucasians by 0.7 (95% CI: 0.2 to 1.2; *p =*.006). However, pre-operative SNB values were lower in Asians compared to Caucasians with a difference of -2.7 (95% CI: -3.9 to -1.5; *p* <.0001). Lastly, postoperative SNB values were higher amongst Caucasians than Asians with a difference of -2.0 (95% CI: -3.1 to -1.0; *p* <.0001).

## Discussion

This is the first systematic review and meta-analysis that discusses racial outcomes in MMA surgery for OSA. Subjective and objective outcomes such as AHI, ESS, LSAT, amount of advancement, SNA, and SNB were measured and compared overall and in different racial groups. This study aimed to evaluate the outcomes of different racial and ethnic groups undergoing MMA for OSA management and to identify potential factors contributing to variations. MMA for OSA was effective for all racial groups, however, there were differences in outcomes and reported data in different racial groups. Among 427 patients, 377 (88.1%) were considered a surgical success. Previous meta-analyses performed also utilized such criteria yet lacked racial data [8, 10]. Additionally, although the Latino group had greater surgical success (100.0%), it was based on one study with 3 Latino patients. Therefore, when accounting for sample size and number of studies, Asians had greater surgical success compared to Caucasians (93.5% vs. 83.2%, respectively). The rates of surgical success in our study for Caucasians were consistent with the surgical success seen in previous meta-analysis (83.2% vs. 85.5%). However, for Asians, surgical success was higher than previous meta-analysis (93.5% vs. 85.5%) [8]. In this review, it was evidenced that Asians had the greatest AHI percent reduction (83.8%) compared to Caucasians and Latinos (76.0% and 71.0%, respectively) groups.

This study is the first to report MMA outcomes for OSA stratified by racial or ethnic groups. The significance of this article lies in the incorporation of cephalometric measures that could explain the encountered racial variation in MMA polysomnographic outcomes. The resulting increase in pharyngeal airway space (PAS) at almost all regions, contributes to improvements in polysomnography [45]. While known for its high success rates post-MMA outcomes in specific racial groups are scarce [8, 10]. Except for Caucasian populations of European ancestry, MMA has not been studied as extensively in other populations (Black, Latinos). This could be attributed to the US population. In 2022 US Census Bureau estimates, 75.5% of the population were Caucasian, 13.6% were Black, 6.3% were Asian, and 19.1% were Latino/Hispanic [46]. Other potential causes of irregular demographic representation in research overall include health literacy, access to care, and educational levels [47, 48]. However, world population estimates reveal that Asians constitute the majority, comprising 60% of the global population [49]. Asians might be reluctant to get MMA surgery/maxillary advancement due to different craniofacial profiles and aesthetic concerns, namely lower nasal dorsum/midfacial hypoplasia [50, 51]. In our study, 52.4% were Caucasians, 46.0% were Asian, and 1.8% were Latino. Most of the individual studies focused on one specific racial population, except for two studies from the senior author which included Caucasians, Asians, and Latinos [9, 21]. For the latter, data analysis was conducted separately for each racial cohort.

Subjective outcomes (ESS) were analyzed separately given the weak correlation with objective measures for OSA [52]. In our study, 284 subjects in 11 studies provided ESS data that could be utilized [9, 20, 29, 34–37, 39, 41, 42]. There was an overall significant improvement in ESS scores, as well as individually by race, except for the Latinos (only 3 patients). Asians had a greater reduction in ESS scores compared to Caucasians, which could be explained by Asian’s lower (worse) LSAT levels preoperatively.

The LSAT is another PSG outcome that negatively correlates with OSA severity [53], and significantly improves after successful treatments like MMA [54]. Overall, there was a significant increase in LSAT [9, 27, 28, 34–39, 41–43]. In individual racial groups, both Caucasians and Asians had a significant improvement in LSAT, with no significant difference in improvements in LSAT between Caucasians compared to Asians.

Cephalometric measurements were analyzed in studies regarding their possible role in OSA subjectively and objectively. Former studies showed PSG improvements especially in AHI amongst patients with increased SNA angles after MMA [55]. This is supported by a former analysis highlighting that maxillary advancement correlates more to AHI reduction [38]. On analyzing each group, we found that SNA increased more in Caucasians compared to Asians. That can be explained by more advancement noted herein. However, there was no significant difference between pre- or postoperative SNA angles amongst Caucasians and Asians. There was no data on SNA angles for Latinos. It is important to take caution when correlating increases in SNA angles with AHI improvements since this was based on 15 patients.

It’s imperative to note that while maxillary advancement, SNA and SNB changes were more (higher/more aggressive) in Caucasians, Asians still showed a more AHI and LSAT improvement. This could be explained by the variability in soft tissue response to skeletal movements amongst different races. Another hypothesis is disease severity at baseline, as highlighted with a worse LSAT (not AHI) in Asians vs. Caucasians herein. The latter could be attributed to the lower SNB found in Asians preoperatively (73.4 degrees), as it was the only lower (deficient) preoperative cephalometric measurement compared to Caucasians. This is supported by former studies highlighting the more retrognathic mandibles and steeper occlusal planes in Asians [56, 57]. While the occlusal plane changes were not reported we could postulate it was more in Asians, given the higher discrepancy between maxillary and mandibular advancements in Asians vs. Caucasians (6.8 vs. 2.8 mm, respectively). This could be another hypothesis explaining superior outcomes in Asians, however, this warrants further prospective investigations.

## Limitations

Most of the studies were very small in sample size, and only contained a handful of subjects of a particular ethnicity, likely making the comparisons difficult. Only a few medical centers have reported their own patients, and this likely could influence the results. Polysomnography measurements (e.g. AHI) vary in definition and in timeline. Studies had a mean follow-up of 8.0 months, however, some did not specify follow-up time [20]. Moreover, out of the 91 studies, only 20 were included in our study due to a lack of racial demographic data [58]. Additionally, the studies included in the analysis did not specify whether AHI 3% or 4% was utilized. Oxygen-desaturation index was not reported. Another limitation is that most of the studies were case-series, and higher-level studies such as case-control, cohort studies, and randomized control trials are not feasible [59]. We also did not include neck circumference and sleep endoscopy results given the absence of data in all articles. There were unknown rates of prognathism/retrognathism, or class 1/2/3 occlusion stratified by race, other than 1 paper by the senior author and that is not sufficient to make a valid analysis. Additionally, even when some included articles reported cephalometric analysis, the patient data is not individualized, (as it is pooled together). Therefore, we can only use means. Lastly, our study featured a substantial sample size of Caucasians and Asians, but only two studies included Latinos, and none included Black patients. This highlights the racial disparity in OSA management particularly with skeletal surgery.

## Conclusion

Maxillomandibular advancement is an effective treatment for OSA in Caucasians, Asians, and Latinos. However, variations in improvement were observed between different racial/ethnic groups; with Asians exhibiting lower initial pre-operative LSAT scores, a greater percent reduction in AHI, and greater surgical success when compared to Caucasians. There is a racial disparity in MMA outcomes amongst Blacks and Latinos, with absent literature in these groups, highlighting potential disparity in OSA treatment as a whole. Future studies should provide individual data regarding maxillary and mandibular advancements to provide insight into whether distinct cephalometric changes are likely to contribute to heightened surgical success rates in patients overall and within individual racial/ethnic groups.

## Electronic supplementary material

Below is the link to the electronic supplementary material.

Supplementary Material 1

Supplementary Material 2

Supplementary Material 3

Supplementary Material 4

Supplementary Material 5

## Data Availability

All extracted raw data regarding this project that support the findings of this study can be requested via email to the corresponding author.
