# Supplementary material for: Racial variations in maxillomandibular advancement for obstructive sleep apnea: a systematic review and meta-analysis
Source: Sleep Breath. 2024 Dec 9;29(1):55. doi: 10.1007/s11325-024-03211-0 (PMC11628450; doi:10.1007/s11325-024-03211-0)
Supplement: Supplementary file 1 — Supplementary Material 1 [file 11325_2024_3211_MOESM1_ESM.docx]

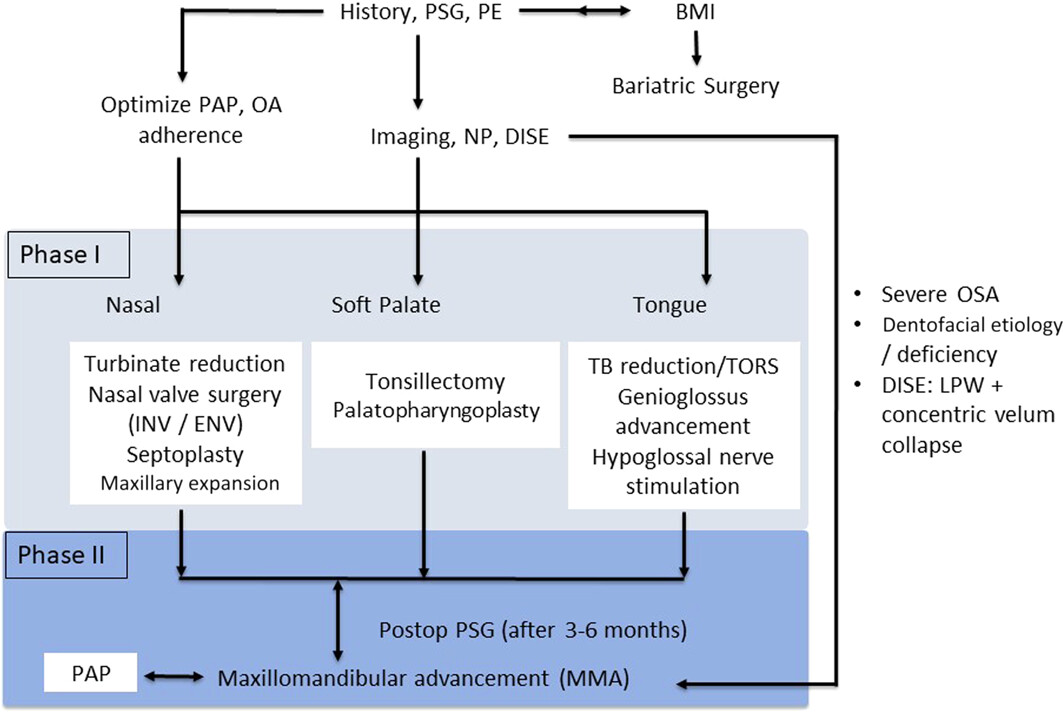


**Supplement 1.** Updated Stanford Sleep Surgery Algorithm. BMI, body mass index; DISE, drug-indued sleep endoscopy; DOME, distraction osteogenesis maxillary expansion; INV/ENV, internal/external valve; LPW, lateral pharyngeal wall; NP, nasopharyngoscopy; OA, oral appliance; OSA, obstructive sleep apnea; PAP, positive airway pressure; PE, physical exam; Postop, postoperative; PSG, polysomnography; TB, tongue-bases; TORS, transoral robotic surgery.
