# Supplementary material for: Racial variations in maxillomandibular advancement for obstructive sleep apnea: a systematic review and meta-analysis
Source: Sleep Breath. 2024 Dec 9;29(1):55. doi: 10.1007/s11325-024-03211-0 (PMC11628450; doi:10.1007/s11325-024-03211-0)
Supplement: Supplementary file 2 — Supplementary Material 2 [file 11325_2024_3211_MOESM2_ESM.docx]

A search was performed from inception until August 8, 2023, in each database. Keywords and phrases included “maxillomandibular advancement”, “double jaw surgery”, “orthognathic surgery”, “maxillary and mandibular osteotomy”, “maxillary and mandibular advancement”, “racial groups”, “race”, “ethnicity, “sleep apnea”, and “obstructive sleep apnea”. One example of a search performed on PubMed is: (Maxillomandibular advancement OR double jaw surgery OR orthognathic surgery OR maxillary and mandibular osteotomy OR maxillary and mandibular advancement) AND ("racial groups"[MeSH Terms] OR race OR ethnicity OR races OR ethnicities OR sleep apnea OR obstructive sleep apnea).

**Supplement 2.** Search Strategy
