# Supplementary material for: Racial variations in maxillomandibular advancement for obstructive sleep apnea: a systematic review and meta-analysis
Source: Sleep Breath. 2024 Dec 9;29(1):55. doi: 10.1007/s11325-024-03211-0 (PMC11628450; doi:10.1007/s11325-024-03211-0)
Supplement: Supplementary file 3 — Supplementary Material 3 [file 11325_2024_3211_MOESM3_ESM.docx]

**Identification of studies via databases and registers**

Records identified from databases (**n=1578**)

PubMed (**n=840**)

Scopus **(n=727)**

CINAHL **(n=11)**

Cochrane **(n=0)**

Records removed *before screening*:

Duplicate records removed via Covidence (**n=519)**

**Identification**

Records screened

**(n=1059)**

Irrelevant studies:

**(n=891)**

Full-text studies assessed for eligibility

**(n=168)**

**Screening**

Reports excluded:

Unable to reach corresponding author for racial/ethnicity data **(n=66)**

Wrong outcomes **(n=23)**

Wrong comparator **(n=13)**

No racial demographic data available after corresponding author email correspondence **(n=9)**

Lack of demographic data **(n=5)**

Wrong intervention **(n=6)**

Wrong patient population **(n=5)**

Full-text unavailable **(n=5)**

Wrong study design **(n=5)**

Abstract only **(n=3)**

non-English text **(n=3)**

Case report **(n=2)**

Discussion only **(n=2)**

Wrong indication **(n=1)**

Reports assessed for eligibility

**(n=168)**

Studies included in review

**(n=20)**

**Included**

**Supplement 3.** PRISMA 2020 flow diagram for new systematic reviews which included searches of databases and registers only.
