# Supplementary material for: Racial variations in maxillomandibular advancement for obstructive sleep apnea: a systematic review and meta-analysis
Source: Sleep Breath. 2024 Dec 9;29(1):55. doi: 10.1007/s11325-024-03211-0 (PMC11628450; doi:10.1007/s11325-024-03211-0)
Supplement: Supplementary file 4 — Supplementary Material 4 [file 11325_2024_3211_MOESM4_ESM.docx]

**Supplement 4.** JBI Critical Appraisal Tool Case Series.

| **Study** | **1** | **2** | **3** | **4** | **5** | **6** | **7** | **8** | **9** | **10** | **Overall** |
| --- | --- | --- | --- | --- | --- | --- | --- | --- | --- | --- | --- |
| **Abdelwahab A 2023** | Y | Y | Y | Y | Y | Y | Y | Y | U | Y | 9, include |
| **Abdelwahab B 2023** | Y | Y | Y | Y | Y | Y | Y | Y | U | Y | 9, include |
| **Bettega 2000** | Y | Y | Y | Y | N | Y | Y | Y | Y | Y | 9, include |
| **Brevi 2015** | Y | Y | Y | Y | N | Y | Y | Y | Y | Y | 9, include |
| **Curran 2022** | Y | Y | Y | Y | Y | Y | Y | Y | N | Y | 9, include |
| **Goh 2003** | Y | Y | Y | Y | Y | Y | Y | Y | Y | Y | 10, include |
| **Gonzalez 2020** | N | Y | Y | U | Y | Y | Y | Y | Y | Y | 8, include |
| **Jeong 2017** | Y | Y | Y | Y | Y | Y | Y | Y | Y | Y | 10, include |
| **Lagana 2023** | Y | Y | Y | Y | Y | Y | Y | Y | Y | Y | 10, include |
| **Liao 2015** | Y | Y | Y | Y | Y | Y | Y | Y | Y | Y | 10, include |
| **Lin 2011** | Y | Y | Y | U | Y | Y | Y | Y | Y | Y | 9, include |
| **Lin 2020** | Y | Y | Y | U | Y | Y | Y | Y | Y | Y | 9, include |
| **Liu 2012** | Y | Y | Y | Y | Y | Y | Y | Y | U | Y | 9, include |
| **Lye 2008** | Y | Y | Y | Y | Y | Y | Y | Y | Y | Y | 10, include |
| **VanderCruyssen 2019** | Y | Y | Y | U | Y | Y | Y | Y | U | Y | 8, include |
| **Varghese 2012** | Y | Y | Y | Y | Y | Y | Y | Y | Y | Y | 10, include |
| **Verze 2017** | Y | Y | Y | U | Y | Y | Y | Y | Y | Y | 9, include |
| **Wei 2017** | Y | Y | Y | U | Y | Y | Y | Y | Y | Y | 9, include |
| **Wu 2019** | Y | Y | Y | Y | Y | Y | Y | Y | Y | Y | 10, include |
| **Yu 2017** | Y | Y | Y | U | Y | Y | Y | Y | Y | Y | 9, include |
| Abbreviations: Y-Yes, N-No, U-Unclear, JBI- Joanna Briggs Institute | | | | | | | | | | | |
| 1. Were there clear criteria for inclusion in the case series? | | | | | | | | | | | |
| 2. Was the condition measured in a standard, reliable way for all participants included in the case series? | | | | | | | | | | | |
| 3. Were valid methods used for identification of the condition for all participants included in the case series? | | | | | | | | | | | |
| 4. Did the case series have consecutive inclusion of participants? | | | | | | | | | | | |
| 5. Did the case series have complete inclusion of participants? | | | | | | | | | | | |
| 6. Was there clear reporting of the demographics of the participants in the study? | | | | | | | | | | | |
| 7. Was there clear reporting of clinical information of the participants? | | | | | | | | | | | |
| 8. Were the outcomes or follow up results of cases clearly reported? | | | | | | | | | | | |
| 9. Was there clear reporting of the presenting site(s)/clinic(s) demographic information? | | | | | | | | | | | |
| 10. Was statistical analysis appropriate? | | | | | | | | | | | |
