# Supplementary figures and images for: Racial variations in maxillomandibular advancement for obstructive sleep apnea: a systematic review and meta-analysis
Source: Sleep Breath. 2024 Dec 9;29(1):55. doi: 10.1007/s11325-024-03211-0 (PMC11628450; doi:10.1007/s11325-024-03211-0)

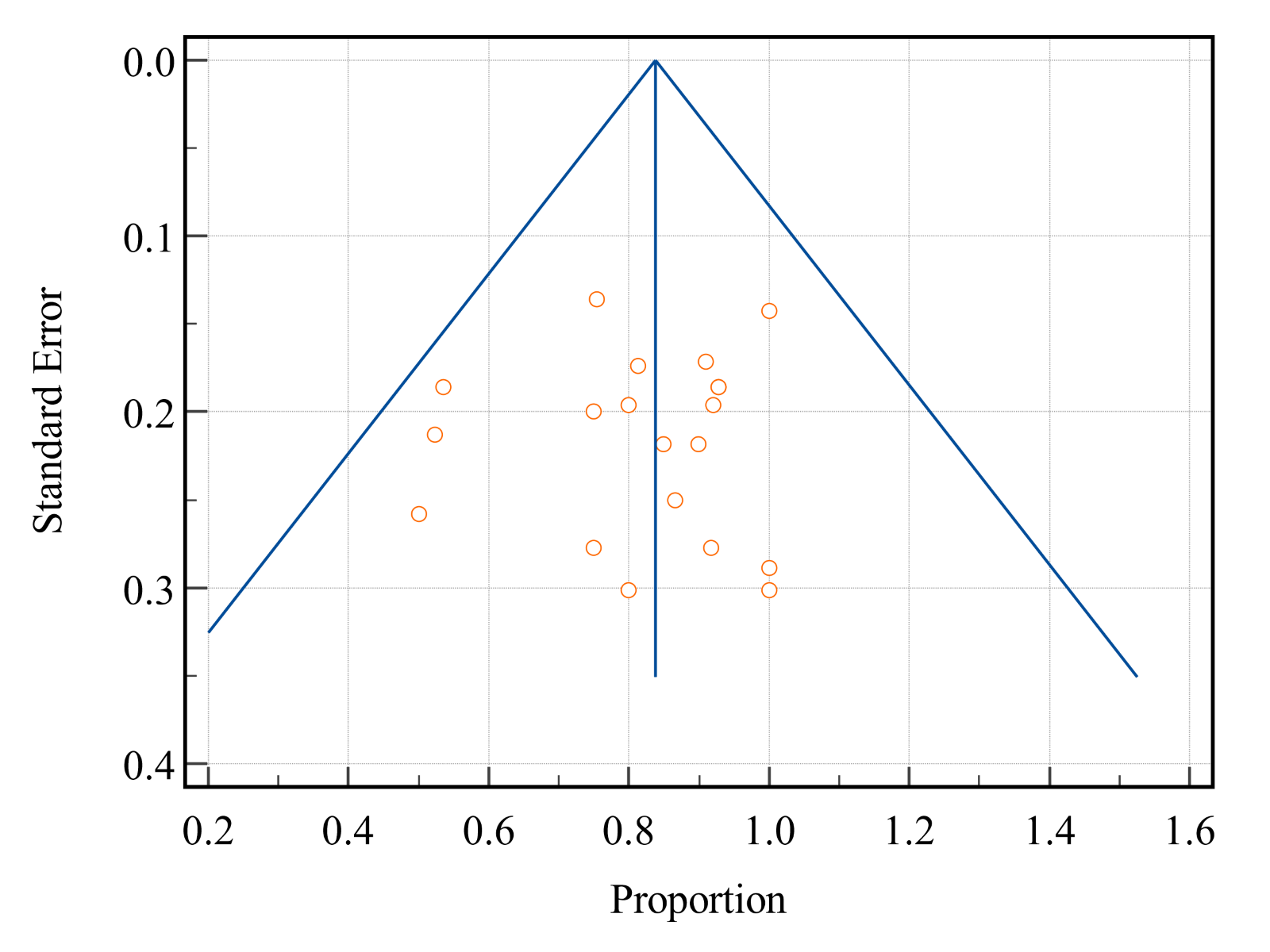
 **Supplement 5.** Egger’s Test for Publication Bias.

Supplement: Supplementary file 5 — Supplementary Material 5 [file 11325_2024_3211_MOESM5_ESM.docx]
